# 84. One-month Humoral Response Following Two Doses of Covid-19 Vaccines in Specific populations – ANRS0001S COV POPART Cohort Study

**DOI:** 10.1093/ofid/ofac492.009

**Published:** 2022-12-15

**Authors:** Paul LOUBET, L I nda Wittkop, Mathieu Chalouni, David-Axel Laplaud, Martine Laville, Bruno Laviolle, Jean-Daniel Lelièvre, Jean-Yves Blay, Benoit Barrou, Benjamin Terrier, Laure Esterle, Stephanie Nguyen, Axel Levier, Eric Tartour, Xavier de Lamballerie, Odile Launay

**Affiliations:** CHU de Nîmes, Nimes, Languedoc-Roussillon, France; Université Bordeaux, Bordeaux, Aquitaine, France; Université Bordeaux, Bordeaux, Aquitaine, France; CHU Nantes, Nantes, Pays de la Loire, France; CHU Lyon, Lyon, Rhone-Alpes, France; CHU Rennes, Rennes, Bretagne, France; CHU Henri Mondor, Creteil, Ile-de-France, France; Unicancer Lyon, Lyon, Rhone-Alpes, France; APHP, Paris, Ile-de-France, France; APHP, Paris, Ile-de-France, France; Université Bordeaux, Bordeaux, Aquitaine, France; APHP, Paris, Ile-de-France, France; ANRS, Paris, Ile-de-France, France; Service d’Immunologie Biologique, APHP, Hôpital Européen Georges Pompidou, 75015 Paris, France; PARCC, INSERM U970, Université de Paris, 75006 Paris, France, Paris, Ile-de-France, France; APHM, Marseille, Provence-Alpes-Cote d'Azur, France; Université Paris Cité; Inserm F-CRIN, I-REIVAC; Assistance Publique Hôpitaux de Paris, Paris, Ile-de-France, France

## Abstract

**Background:**

We evaluated the immune response to COVID-19 vaccines in several specific populations at high risk of severe COVID-19.

**Methods:**

Participants from the French national multi-center prospective cohort study ANRS0001S COV-POPART were included (11 specific subpopulations: and 2 control groups (18–64 years and over 65 years)). In this preliminary analysis patients and controls who had received at least two vaccine doses have been included. Percentages (95% confidence intervals (CI)) of participants with anti-Spike SARS-CoV-2 IgG antibodies (ELISA) and specific neutralizing antibodies (*in vitro* neutralization assay) were evaluated at one month after the second dose of COVID-19 vaccine.

**Results:**

3703 were included: 2650 participants from specific subpopulations (171 solid cancers, 160 SOT, 100 HCT, 91 chronic renal failures, 141 systemic autoimmune diseases, 157 autoimmune inflammatory rheumatic diseases, 361 multiple sclerosis (MS) or neuromyelitis optica spectrum disorders, 61 hypogammaglobulinemia, 401 diabetic, 739 obeses non-diabetic and 476 HIV) and 1053 controls (893: 18–64 years and 160 over 65 years). Median age was 51.7 years [InterQuartile range: 40.8 – 60.9] and 50.7% were male. Most of the participants received BNT162b2 vaccine (86.4%). In the control group, 100% (95%CI: 99.6;100.0) of those aged 18–64 and 99.4% (96.6; 100.0) of those over 65 years developed anti-Spike IgG antibodies.

PLWHIV, cancer and diabetic patients had high rate of responders after two doses with 98.3% (97.2;99.1), 93.0% (88.1;96.3) and 92.0% (88.9;94.5), respectively. The lowest percentage of responders was found in patients with SOT (13.8% (8.8;20.1), HSCT (34.0% (24.8;44.2) and hypogammaglobulinemia (52.5% 39.3;65.4). In both control groups, the frequency of neutralizing antibodies was similar to the anti-Spike IgG antibody response. In the immunodeficient populations, neutralizing antibodies responders tended to be less frequent than anti-Spike antibodies responders. Similar trends than for IgG antibody were identified (Figure 1).

Anti-Spike and Neutralizing antibody (Ab) responses (95% CI) one month after the second dose of COVID-19 vaccine in specific and control populations.

**Conclusion:**

Lower COVID-19 vaccine humoral response was observed in specific populations than in controls, especially in patients with hypogammaglobulinemia, HSCT and SOT.

**Disclosures:**

**Paul LOUBET, MD, PhD**, pfizer: Board Member **Odile Launay, MD, PhD**, AstraZeneca: Financial|GlaxoSmithKline: Advisor/Consultant|GlaxoSmithKline: Grant/Research Support|Johnson & Johnson: Advisor/Consultant|Johnson & Johnson: Grant/Research Support|MD: Advisor/Consultant|Moderna: Advisor/Consultant|MSD: Data safety monitoring board|Pfizer: Advisor/Consultant|Pfizer: Grant/Research Support|Sanofi Pasteur: Advisor/Consultant|Sanofi Pasteur: Grant/Research Support|Sanofi Pasteur: Data safety monitoring board.

